# Transcriptome- and proteome-wide effects of a circular RNA encompassing four early exons of the spinal muscular atrophy genes

**DOI:** 10.21203/rs.3.rs-3818622/v1

**Published:** 2024-02-28

**Authors:** Diou Luo, Eric Ottesen, Ji Heon Lee, Ravindra Singh

**Affiliations:** Iowa State University; Iowa State University; Iowa State University; Iowa State University

**Keywords:** spinal muscular atrophy, SMA, Survival Motor Neuron, SMN, circular RNA, circRNA, proteome, transcriptome

## Abstract

Spinal muscular atrophy (SMA) genes, *SMN1* and *SMN2*, produce multiple circular RNAs (circRNAs), including C2A-2B-3–4 that encompasses early exons 2A, 2B, 3 and 4. Here we report the transcriptome- and proteome-wide effects of overexpression of C2A-2B-3–4 in inducible HEK293 cells. Our RNA-Seq analysis revealed altered expression of ~ 15% genes (4,172 genes) by C2A-2B-3–4. About half of the affected genes by C2A-2B-3–4 remained unaffected by L2A-2B-3–4, a linear transcript encompassing exons 2A, 2B, 3 and 4 of *SMN1/SMN2*. These fifindings underscore the unique role of the structural context of C2A-2B-3–4 in gene regulation. A surprisingly high number of upregulated genes by C2A-2B-3–4 were located on chromosomes 4 and 7, whereas many of the downregulated genes were located on chromosomes 10 and X. Supporting a cross-regulation of *SMN1/SMN2* transcripts, C2A-2B-3–4 and L2A-2B-3–4 upregulated and downregulated *SMN1/SMN2* mRNAs, respectively. Proteome analysis revealed 61 upregulated and 57 downregulated proteins by C2A-2B-3–4 with very limited overlap with those affected by L2A-2B-3–4. Independent validations confirmed the effect of C2A-2B-3–4 on expression of genes associated with chromatin remodeling, transcription, spliceosome function, ribosome biogenesis, lipid metabolism, cytoskeletal formation, cell proliferation and neuromuscular junction formation. Our findings reveal a broad role of C2A-2B-3–4, a universally expressed circRNA produced by *SMN1/SMN2*.

## INTRODUCTION

Circular RNAs (circRNAs) are produced in cells of all living organisms^1–3^. Due to absence of free termini, circRNAs are more stable than the linear RNAs[Bibr R4]. Generation of circRNAs usually involves backsplicing in which a downstream 5′ splice site (5′ss) pairs with an upstream 3′ss^5,6^. Biogenesis of circRNAs is a tightly controlled process as backsplicing competes with forward splicing, which is responsible for the production of linear transcripts[Bibr R7]. Splice site pairing for backsplicing is facilitated by RNA structures as well as by RNA-binding proteins[Bibr R6]. RNA structures formed by inverted repeat sequences, including Alu elements favor generation of circRNAs^8–10^. Considering Alu elements are present only in primates and are continuing to evolve, their presence within intronic sequences provide species-specific mechanisms for the generation of circRNAs[Bibr R11]. In some instances, Alu-independent RNA structures enabled by long-range base pairing play pivotal role in regulation of pre-mRNA splicing, including backsplicing[Bibr R12]. Functions of circRNAs include sequestration of proteins, sponging of microRNAs, transcription regulation and production of novel proteins^13–16^. CircRNAs are associated with a growing number of pathological conditions and offer novel avenues for diagnosis and therapy^17–19^.

Spinal Muscular Atrophy (SMA) is one of the leading genetic causes of infant mortality^20–23^. In more than 95% cases, SMA results from the deficiency of Survival Motor Neuron (SMN) protein due to deletions of or mutations in *SMN1* gene^20–23^. SMN is involved in many functions, including DNA repair, transcription, translation, mRNA trafficking, stress granule formation and cell signaling[Bibr R24]. *SMN2*, a near identical copy of *SMN1*, fails to compensate for the loss of *SMN1* due to predominant skipping of exon 7^25,26^. Transcripts lacking exon 7 code for SMNΔ7, a less stable and partially functional protein[Bibr R27].

Restoration of SMN by promotion of *SMN2* exon 7 inclusion or by gene therapy are the proven approaches for the treatment of SMA^22,28–31^. Deficiency of SMN has also been shown to cause male reproductive organ developmental defects in mice[Bibr R32]. In contrast, overexpression of SMN has been found to trigger neuroinflammation and the innate immune response in mice[Bibr R33]. Recently, overexpression of SMN is recorded in patients with laryngeal squamous cell carcinoma (LSCC)[Bibr R34]. Hence, a tight regulation of SMN appears to be critical for the proper function of the cellular metabolism.

Mechanisms by which *SMN1/SMN2* genes modulate SMN levels involve both transcription and splicing regulation[Bibr R35]. About 40% of the sequences of human *SMN* genes are comprised of Alu elements with the potential to exert unique transcriptional and post-transcriptional regulations[Bibr R36]. One of the Alu elements of *SMN1/SMN2* encompasses the alternatively spliced exon 6B, inclusion of which changes the critical C-terminus of SMN[Bibr R37]. However, exon 6B of *SMN1/SMN2* is generally skipped and the exon 6B-included transcripts are subjected to nonsense-mediated decay (NMD)[Bibr R37]. Consistent with the unusually high number of Alu elements within intronic sequences, human *SMN1/SMN2* genes generate a huge repertoire of circRNAs^38,39^. DNA/RNA helicase DHX9 (also known as RHA) and splicing factor Sam68 have been shown to modulate generation of *SMN1/SMN2* circRNAs^38,39^. DHX9 is a SMN-interacting protein and is a known regulator of transcription and splicing^40–42^. Sam68 has been previously implicated in regulation of *SMN2* exon 7 splicing[Bibr R43]. Recent evidence supports that the process of forward splicing and/or the presence of the exon junction complex (EJC) formed during forward splicing favor generation of *SMN1/SMN2* circRNAs[Bibr R44]. Considering pre-mRNAs serve as the source for both circRNAs and mRNAs, generation of *SMN1/SMN2* circRNAs may have a role in modulating SMN levels by reducing the levels of *SMN1/SMN2* mRNAs. Another way by which *SMN* circRNAs may modulate SMN levels is through sequestration of microRNAs (miRNAs). However, functions of *SMN* circRNAs remain largely unknown.

**Figure 1:**
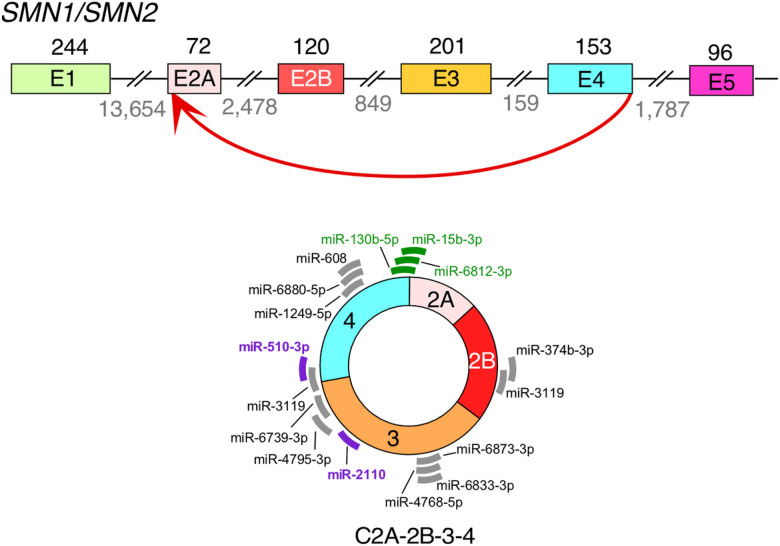
An overview of backsplicing event to form C2A-2B-3–4 from *SMN1/SMN2* genes. Top panel: Genomic overview of the early exons of *SMN1/SMN2* genes. Exons are depicted in colored shapes and introns in broken lines. Sizes of exons and introns are marked above exons and below introns, respectively. The backsplicing event to form C2A-2B-3–4 is marked with a red arrow. Bottom panel: A diagram of C2A-2B-3–4. Predicted miRNA binding sites are indicated with thick lines [Bibr R45]. Gray color represents miRNAs of unknown function. Purple color represents miRNAs with identified functions related to SMN biology and/or neurodegeneration. Green color represents miRNAs whose targets are located across the backsplice junction. Thus, these miRNAs bind differentially to circRNA as compared to their linear mRNA counterparts.

The 546-nucleotide long C2A-2B-3–4 is an abundantly expressed *SMN1/SMN2* circRNA encompassing early exons 2A, 2B, 3 and 4 (Fig. 1)[Bibr R38]. C2A-2B-3–4 is expressed in all human tissues examined, including brain, spinal cord, heart, skeletal muscle, smooth muscle, liver, kidney, lung, uterus and testis[Bibr R38]. C2A-2B-3–4 is also expressed in mouse, underscoring its functional significance across the mammalian kingdom[Bibr R38]. C2A-2B-3–4 is predicted to interact with at least 16 miRNAs, three of which specifically target the backsplice junction (miR-130b-5p, miR-6812–3p, and miR-15b-3p) and two of which are associated with SMN-related biology and/or neurodegenerative diseases (miR-2110 and miR-510–3p)[Bibr R45]. We recently reported HEK293-derived inducible stable-cell-lines TC4–2A and TL4–2A that express C2A-2B-3–4 and L2A-2B-3–4, respectively[Bibr R44]. L2A-2B-3–4 is a linear transcript encompassing exons 2A, 2B, 3 and 4 of *SMN1/SMN2*. Here we employ TC4–2A and TL4–2A cells to examine the effect of overexpression of C2A-2B-3–4 and L2A-2B-3–4, receptively, on transcriptome and proteome. Our findings reveal role of C2A-2B-3–4 in regulation of transcription, spliceosome function, ribosome biogenesis, lipid metabolism, cytoskeletal formation, and cell proliferation. Expressions of many genes associated with neuronal functions were also affected by C2A-2B-3–4. Our report expands the functions of *SMN1/SMN2* genes in regulation of diverse cellular processes.

## MATERIALS AND METHODS

### Mammalian cell culture

Abbreviations used in this publication are listed in Supplementary Table S1. HEK293-derived stable cell lines TC4–2A and TL4–2A expressing C2A-2B-3–4 and L2A-2B-3–4, receptively, used here were previously generated from the commercially available T-REx cells[Bibr R44]. TC4–2A and TL4–2A cells were cultured in Dulbecco’s modified Eagle’s medium (DMEM, Gibco, Cat No. 11965–092), supplemented with 10% fetal bovine serum (Gibco, Cat No. 16000–044) and 1× Glutamax (Gibco, Cat No. 35050061). Antibiotics were added following the manufacturers’ instruction and/or experimentally determined values, including 100 μg/mL zeocin, 15 μg/mL blasiticidin, 100 μg/mL hygromycin (Life technologies, Cat No. R250–01, R210–01, R220–05) and 0.1 μg/mL doxycycline (Dox, Sigma-Aldrich, Cat No. D9891–10G). Cells were cultured in CO_2_ incubator (Thermo Fisher Scientific, NAPCO Series 8000WJ) at 37 °C and 5% CO_2_. For induction of expression, TC4–2A, TL4–2A and control T-REx cells were seeded at a density of 2 × 10^5^ cells per 100 mm dish. After incubating for 18 hours (h), the cell culture medium was replaced with fresh medium containing Dox. After 48 h of incubation, the cell culture medium was replaced with fresh medium containing Dox. The cells were collected 48 h after the second addition of Dox (96 h of induction in total).

### RNA isolation and RNase R treatment

Total RNA was isolated from cells using TRIzol reagent (Invitrogen, Cat No. 15596018). RNA was digested with RQ1 RNase-free DNase (Promega, Cat No. M6101) to remove contaminating genomic DNA, followed by phenol:chloroform extraction and ethanol precipitation. Then RNA was treated with RNase R (Applied Biological Material Inc., Cat No. E049), a 3¢-to-5 ¢ exoribonuclease to eliminate linear transcripts. RNase R treatment was performed in 10 μL reaction, in which 0.5 μL (10 U) of RNase R was used to treat 2 μg of RNA. The treatment was performed for 45 min (min) at 37 °C, followed by inactivation at 65 °C for 20 min. To estimate the digestion efficiency, mock reactions without enzyme were carried out side by side.

### Reverse transcription and PCR (RT-PCR)

Superscript III reverse transcriptase (RT) (Invitrogen, Cat No. 18080044) was used in cDNA synthesis following the manufacturer’s instruction. Per 5 μL RT reaction, 0.5 μg of total RNA was used. Reactions were primed with either a gene-specific or a random primer (Promega, Cat No. C1181) (Supplementary Table S2). For semi-quantitative PCR, 1 μL of cDNA was added per 20 μL reaction. PCR products were separated by electrophoresis on 6% native polyacrylamide gels and visualized by ethidium bromide staining. For qPCR, 3 μL diluted cDNA (1:20 dilution) was used in a 20 μL qPCR reaction containing 1× PowerUp SYBR green master mix (Life Technologies, Cat No. A25742) and the desired primer set. Biological triplicates of qPCR reactions were performed on a QuantStudio 3 thermocycler (Thermo Fisher Scientific). Relative expression was determined using the 2^−ΔΔCt^ method using *GAPDH* as housekeeping gene. To determine the copy number of circRNA per cell, we used a standard curve defined by serial dilution of a known quantity of linearized plasmid containing the sequence of the expected PCR product[Bibr R44]. All primers were obtained from Integrated DNA Technologies. Primers used in this publication are listed in Supplementary Table S2.

### Library Generation and RNA-Seq

To confirm RNA integrity, TRIzol-isolated total RNA was characterized using an Agilent Bioanalyzer on an RNA nano chip (RIN ≥8). 1 μg of total RNA was then subjected to rRNA depletion using the NEBNext rRNA depletion kit v2 (Human/Mouse/Rat). Libraries were generated from rRNA-depleted RNA using the NEBNext Ultra II directional RNA library prep kit for Illumina. Libraries were barcoded for multiplexing using NEBNext Dual Index oligos for Illumina. Size distribution of libraries was determined using an Agilent Bioanalyzer DNA 1000 chip and quantified using a Qubit uorimeter. Libraries were pooled together and sequenced on an Illumina Novaseq 6000 using an S23 ow cell following a 100-cycle, paired-end protocol. Reads from RNA-Seq were mapped to the human reference genome build GRCh38 using HISAT2[Bibr R46]. For differential expression, mapped reads were assigned to genes according to the Gencode v33 human transcriptome annotation[Bibr R47] using the featureCounts script from the Subread software package[Bibr R48]. Differential expression was estimated using the DESeq2 R package[Bibr R49]. RNA-Seq data is available from the NCBI sequence read archive under project number PRJNA1058619.

### Protein lysate preparation

Cells were washed in ice-cold phosphate buffered saline (PBS) for three times and collected by scraping in 1 mL PBS followed by centrifugation at × 2500 g for 2 min. PBS supernatants were aspirated, and pelleted cells were lysed using lysis buffer, which contained 500 mM triethylammonium bicarbonate (TeABC, Sigma–Aldrich, Cat No. 18597–100ML), 1% sodium deoxycholate (SDC, Sigma–Aldrich, Cat No. D5670–5G), and 1× Halt^™^ Protease, Phosphatase Inhibitor Cocktail (Thermo Fisher Scientific, 100X, Cat No. 78440)[Bibr R50]. The lysates were incubated on ice for 45 min, and then sonicated. Samples were centrifuged at 16,000 × g for 30 min at 4 °C. Clear supernatants were collected and transferred to new tubes. The puri ed protein was snap-frozen on dry ice and stored at −80 °C. Protein concentrations were measured by Bio-Rad protein assay (Bio-Rad, Cat No. 5000006).

### Label-free proteomic quantification and analysis

About 50 μg crude protein samples were submitted to the Protein Facility of the Iowa State University O ce of Biotechnology (https://www.protein.iastate.edu/). The samples were processed by a label-free relative quantification approach, using the Minora Feature Detector to detect and quantify isotopic clusters. Specifically, the samples were digested with trypsin/Lys-C. SDC was removed by acid precipitation with 2% (v/v) trifluoroacetic acid (TFA). Then, PRTC standard (Pierce Biotechnology, Cat No. 88320) was spiked in to serve as an internal control. The fragmented peptides were then separated by liquid chromatography with tandem mass spectrometry (LC-MS/MS), using the Q Exactive^™^ Hybrid Quadrupole-Orbitrap Mass Spectrometer system (Thermo Fisher Scientific). The relative abundance of the protein was quantified using peak-area based quantification of the precursor ions of the top three most abundant peptides from the identified protein. Then the raw data was computed using Proteome Discoverer Software (Thermo Fisher Scientific, Version 2.4). The data was searched against Mascot and/or Sequest HT. Common contaminants in LC-MS/MS were excluded for downstream analysis^51,52^. MetaboAnalyst platform (https://www.metaboanalyst.ca/, Version 5.0) was used for statistical and bioinformatics analysis, and generation of plots [Bibr R53]. Features with more than 50% missing values were removed. Interquartile range (IQR) was used for data filtering. Data was normalized by quantile normalization and log transformation. Enrichment analysis was performed by WEB-based GEne SeT AnaLysis Toolkit (http://www.webgestalt.org/, Version 2019, accessed in 2022 December)[Bibr R54]. Analysis included Gene Ontology terms (GO terms) for biological processes, cellular components and molecular functions. Pathways including those associated with core protein complex subunits, diseases, phenotypes and the chromosomal locations were defined by Kyoto Encyclopedia of Genes and Genomes (KEGG), Panther, Reactome and Wikipathways. The mass spectrometry proteomics data have been deposited to the ProteomeXchange Consortium with identi er PXD048160.

### Western blot

A total amount of 10–50 μg of protein was loaded per lane of a 10% SDS-PAGE gel. Proteins were transferred from gel to a PVDF membrane using a Transblot Turbo fast transfer system (Bio-Rad) after electrophoresis. To block the blots, 5% non-fat milk dissolved in Tris-buffered saline containing 0.05% Tween-20 (TBST) was used. Primary antibody incubation was performed at 4 °C overnight with gentle agitation. The dilutions of primary antibodies were prepared as follows: mouse anti-α-tubulin 1:4000 (Sigma-Aldrich, Cat No. T6199), mouse anti-CNDP2 1:500 (Proteintech, Cat No. 14925–1-AP), mouse anti-NAMPT/PBEF (Santa Cruz, sc-166946) 1:500, rabbit anti-H1–10 (also known as H1x, Fortis Life Sciences, Cat No. A304–604A-T) 1:2,500. After primary antibody incubation, blots were washed in TBST for 10 min and repeated for a total of three times. Then the blots were incubated with secondary antibodies for 1 h at room temperature, with gentle agitation. The preparation of secondary antibody dilutions was as follows: goat anti-mouse 1:4000 (Jackson ImmunoResearch Laboratories Inc, Cat No. 115-035-003), donkey anti-rabbit 1:2000 (GE Healthcare, Cat No. NA934). After secondary antibody incubation, blots were washed in TBST for 10 min three times and developed using Clarity Western ECL Substrate (Bio-Rad, Cat No. 1705061) or SuperSignal West Femto Maximum Sensitivity Substrate (Thermo Fisher Scientific, Cat No. 34094). The UVP Biospectrum AC imaging system was used to visualize the bands. Quantification of band intensity was performed by using ImageJ software.

### Statistical analysis

Excel (Microsoft, Version 16.62) was used for all the calculation and generation of plots. Data were expressed as mean ± SEM. The unpaired Student’s t-test was applied in statistical analysis. Unless otherwise mentioned, experiments were performed in triplicate, and *p* values were two-tailed and the level of statistical significance was set as *p* < 0.05.

## RESULTS

### Transcriptome-wide effect of overexpression of C2A-2B-3–4

**Figure 2:**
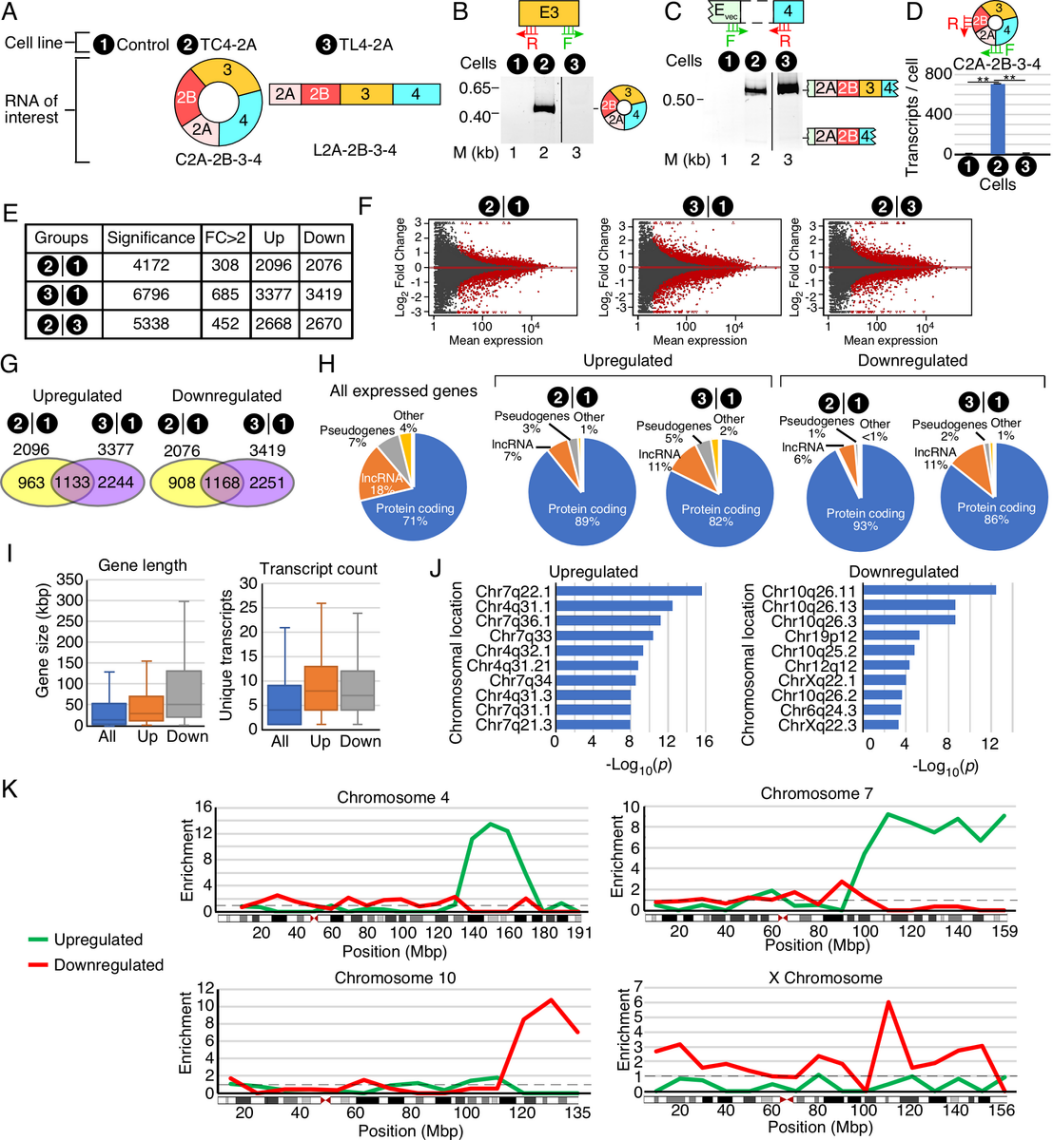
The effect of overexpression of SMN C2A-2B-3–4 on the transcriptome. **(A)** Diagram showing overexpression of C2A-2B-3–4 and L2A-2B-3–4 from TC4–2A and TL4–2A cells, respectively. Control T-REx cells do not overexpress *SMN1/SMN2* transcripts. **(B)** A representative gel showing the results of semi-quantitative PCR for detection circRNA C2A-2B-3–4. Primer annealing sites and cell lines are shown on the top of gel, while lane number is indicated at the bottom. The size marker (M) is indicated on the left side of gel, while the identity of each band on the right side. (C) Identification of vector-specific linear transcripts of *SMN1/SMN2* generated in different cells. Labeling is the same as in (B). **(D)** Quantification of C2A-2B-3–4 expression by qPCR. A diagrammatic representation of circRNA of interest and primer annealing sites are indicated on the top of bar graph. The bar graph shows the number of C2A-2B-3–4 transcripts per cell as determined by qPCR. Error bars represent standard error of the mean. Statistical significance: **, *p*<0.01. **(E)** Summary of RNA-Seq measuring the impact of overexpression of C2A-2B-3–4 and its linear counterpart L2A-2B-3–4 on the transcriptome. “Significant” indicates genes with Benjamini and Hochberg adjusted *p* value (adj. p) < 0.05, out of 19,025 total genes examined for differential expression. “FC >2” indicates genes with more than 2-fold up- or downregulation. **(F)** MA plots depicting gene expression changes upon overexpression of C2A-2B-3–4 or L2A-2B-3–4, or a direct comparison between the two. Y axis: log2 fold change. X axis: mean normalized read counts per gene. Red dots indicate genes with significantly altered expression. **(G)** Venn diagrams examining the overlap between genes altered in the induced TC4–2A and TL4–2A cells compared to control, with upregulated (left panel) and downregulated (right panel) genes indicated. Cell types along with the total number of affected genes are indicated at the top. **(H)** Proportion of protein-coding genes, pseudogenes and lncRNAs among differentially affected genes in TC4–2A and TL4–2A cells compared to T-REx control **(I)** Box plots summarizing the distributions of gene length (left panel) and transcript count (right panel). Boxed area shows the interquartile range (IQR) spanning the middle 50% of values for the given quantification. The median is indicated with a line. Whiskers above and below the box represent upper and lower bound, minus outliers. **(J)** Over-representation analysis (ORA) for specific chromosomal locations of upregulated and downregulated genes. Genomic regions are indicated at the left of each graph. X axis: −log_10_ of *p* values of enrichment. Left panel examines enrichment in genes upregulated by TC4–2A overexpression, right panel downregulated genes. **(K)** Enrichment by chromosomal position for four chromosomes identified in ORA. Y axis: enrichment for a given chromosomal region in upregulated (green) or downregulated (red) genes as compared to all genes expressed in T-REx cells. Dashed line indicates enrichment ratio of 1.0, or average enrichment. X axis: chromosomal position in million base pairs (Mbp). Genes were assigned based on their start position and sorted into bins of 10 Mbp. A graphical overview of each chromosome is depicted below each graph. Boxes indicate chromosomal regions, and red triangles indicate centromere position.

To determine the transcriptome-wide effect of overexpression of C2A-2B-3–4, a circRNA harboring *SMN1/SMN2* exons 2A, 2B, 3 and 4, we employed the recently reported inducible TC4–2A cell line[Bibr R44]. For the purposes of comparison, we also employed inducible TL4–2A cell line that overexpresses L2A-2B-3–4, a linear transcript harboring *SMN1/SMN2* exons 2A, 2B, 3 and 4 (Fig. 2A)[Bibr R44]. As a control, we used T-REx cell line. Overexpression of C2A-2B-3–4 and L2A-2B-3–4 was induced by treatment of the corresponding cell lines with Dox for 96 h. We validated overexpression by semi-quantitative PCR (Figs. 2B–2C). Of note, we used only 22 and 25 cycles of PCR to capture overexpression of C2A-2B-3–4 and L2A-2B-3–4 in TC4–2A and TL4–2A cells, respectively. We also captured linear transcripts (L2A-2B-3–4) as precursors of circular transcripts (C2A-2B-3–4) in TC4–2A cells. In addition, we observed a minor exon 3-skipped transcript L2A-2B-4, which showed a slightly higher expression in TC4–2A cells than in TL4–2A cells. C2A-2B-3–4 was not detected in either TL4–2A cells or in control T-REx cells at 22–25 cycles of PCR amplification. To accurately quantify the expression of C2A-2B-3–4, we performed qPCR using a specific primer annealing to the backsplice junction of C2A-2B-3–4 (Fig. 2D). TC4–2A produced an estimated 708 copies of C2A-2B-3–4 per cell, while less than 0.01 copies per cell were produced in TL4–2A and control T-REx cells. Upon confirming the overexpression of C2A-2B-3–4 and L2A-2B-3–4 in induced TC4–2A and induced TL4–2A cells, respectively, we performed RNA-Seq on transcripts of these cells. Compared to the control T-REx cells, TC4–2A and TL4–2A cells showed altered expression of 4172 and 6796 transcripts, respectively, with 308 and 685 genes undergoing major changes of more than 2-fold, respectively, (Fig. 2E). As a frame of reference, we analyzed 26,828 genes with measurable expression in T-REx cells, meaning ~ 16% and ~ 25% of transcripts were affected in TC4–2A and TL4–2A cells, respectively. Altered expression was evenly split between upregulated and downregulated transcripts (Figs. 2E, F). ~55% of genes affected in TC4–2A cells were similarly affected in TL4–2A cells (Fig. 2G). Among upregulated genes, this amounted to a 4.3-fold enrichment compared to random chance as calculated by a hypergeometric test (*p* = 2.4×10^−516^). For downregulated genes, there was a 4.4-fold enrichment (*p* = 7.6×10^−554^). The remaining 45% genes with altered expressions were unique to TC4–2A cells (Fig. 2G). Direct comparison between transcripts of TC4–2A and TL4–2A cells showed altered expressions of 5338 genes (Figs. 2E, F). We observed disproportionately affected protein-coding genes in both TC4–2A and TL4–2A cells, with lncRNA and pseudogenes under-represented in both upregulated and downregulated genes compared to the parent set of 26,828 genes expressed in T-REx cells.

For the remainder of our analyses, we focused on the genes specifically affected in TC4–2A cells overexpressing C2A-2B-3–4. Upregulated genes were lofinger on average, with a median size of 30.4 kilobases (kb) compared to 14.8 kb for all genes expressed in T-REx cells (Fig. 2I, left panel). Downregulated genes were even lofinger, with a median size of 50.5 kb. Affected genes had higher transcript complexity with a median unique transcript count of 8 and 7 for upregulated and downregulated genes, respectively, compared to 4 for all genes (Fig. 2I, right panel). There was a marked enrichment in upregulated genes for several regions in chromosomes 4 and 7, and for downregulated genes in several regions of chromosomes 10 and X (Fig. 2I). We mapped the genes identified by RNA-Seq by chromosomal location and calculated enrichment throughout chromosomes 4 and 7 (Fig. 2J). We found 61 upregulated genes located between 135 megabase (Mb) and 160 Mb on chromosome 4, representing ~ 46% of all expressed genes in the same region, a 12.9-fold enrichment over a random distribution. There were no downregulated genes in the same region of chromosome 4. We identified 187 upregulated genes located between 90 Mb and the end of the chromosome 7 (~ 159 Mb), representing ~ 28% of all expressed genes in the same region, a 7.8-fold enrichment. Downregulated genes in this region of chromosome 7 were under-represented by 3.2-fold. There were 62 downregulated genes from 110 Mb to the end of chromosome 10 (~ 133 Mb), representing ~ 30% of all expressed genes located in the same region, a 9-fold enrichment (Figs. 2I, J). There were no downregulated genes in the same region of chromosome 10. We observed moderate enrichment of downregulated genes across the entire X chromosome (Fig. 2J). Specifically, there were 65 downregulated genes, ~ 8% of all X-linked genes, representing 2.3-fold enrichment. Upregulated genes were under-represented by 1.9-fold. The region from 100 Mb to 115 Mb of the X chromosome was the most highly enriched, which harbored 26 downregulated genes. This accounted for 20% of all expressed genes in this region, a 6-fold enrichment. Upregulated genes in this same region were under-represented by ~ 1.5-fold.

### Gene ontology terms and pathways affected by overexpression of C2A-2B-3–4

**Figure 3:**
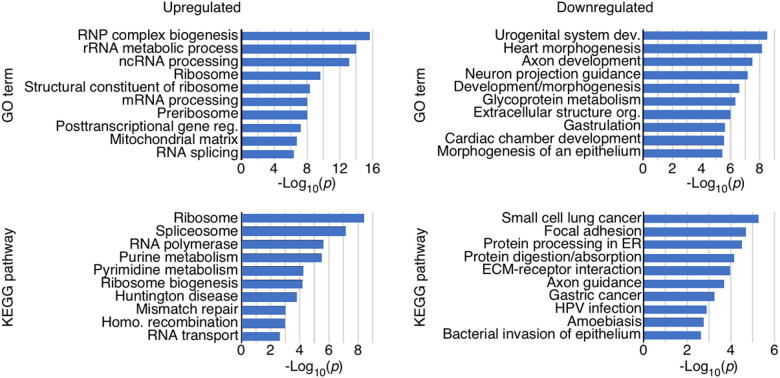
Over-representation analysis (ORA) of GO terms and KEGG pathways. ORA for specific GO terms and Kyoto Encyclopedia of Genes and Genomes (KEGG) pathways. Categories of genes are indicated at the left of each graph. X axis: −log_10_ of *p* values of enrichment. Left panels examine enrichment in genes upregulated by overexpression of C2A-2B-3–4, right panels downregulated genes by C2A-2B-3–4.

Gene ontology (GO) terms group genes by functional category within three groupings: biological process, cellular component, and molecular function. Genes upregulated in TC4–2A cells overexpressing C2A-2B-3–4 were enriched for several GO terms related to RNA regulation and ribonucleoprotein (RNP) biogenesis, suggesting a major shift in RNA regulation and metabolism (Fig. 3). In contrast, genes downregulated in TC4–2A cells were enriched for GO terms related to the extracellular matrix, and cellular morphology (Fig. 3). We also analyzed genes for enrichment in functional pathways as defined by the Kyoto Encyclopedia of Genes and Genomes (KEGG). Upregulated genes were enriched for several pathways involved in RNA regulation, including RNA polymerase, spliceosome, ribosome, and RNA transport. We observed an enrichment in the pathways for homologous recombination and DNA mismatch repair, suggesting an effect on DNA maintenance (Fig. 3). Downregulated genes were enriched for pathways involving protein processing, endoplasmic reticulum (ER), and cell morphology and motility.

### Validation of a broad spectrum of genes upregulated by overexpression of C2A-2B-3–4

**Figure 4:**
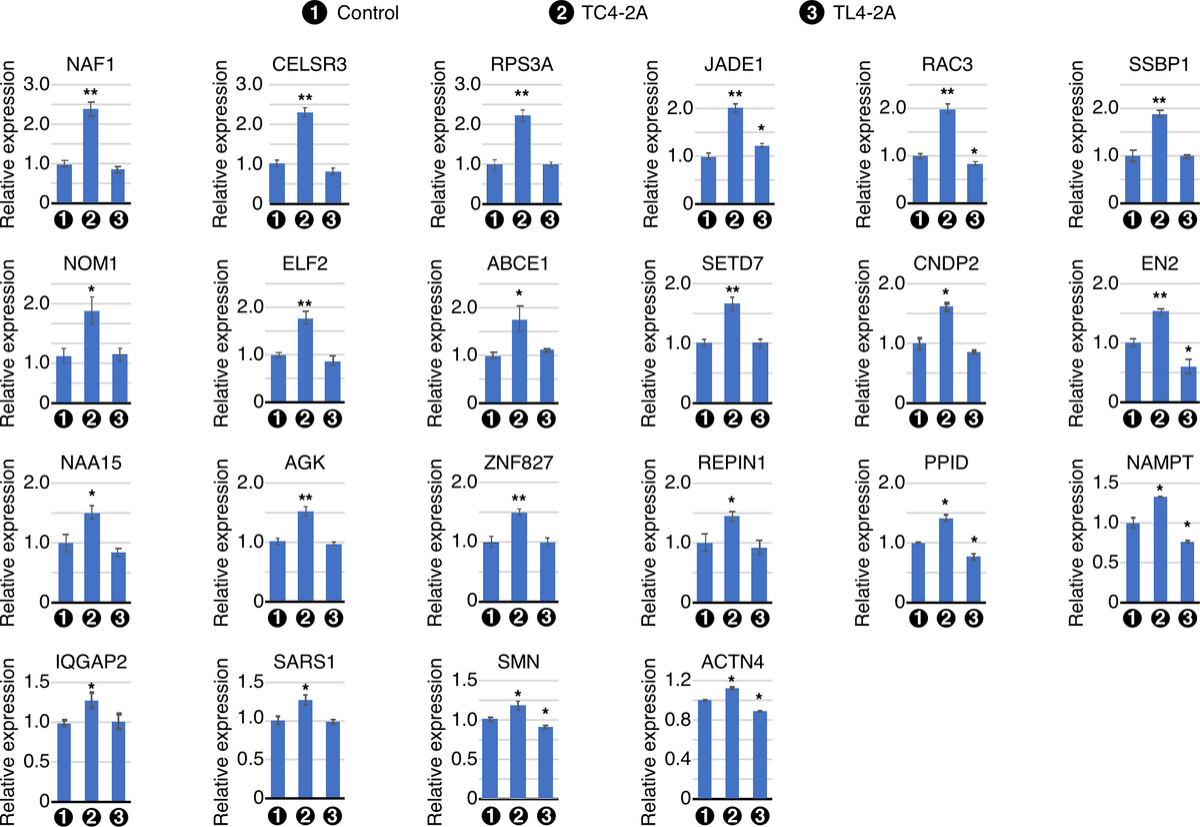
Validation of genes upregulated in RNA-seq. qPCR quantification of mRNA expression levels of significantly upregulated genes upon overexpression of C2A-2B-3–4 is shown in bar graphs. The gene symbol is indicated above each graph. Cell types are indicated under the X-axis. The Y -axis represents the relative expression as compared with control T-REx cells. Error bars represent standard error of the mean. Statistical significance: n = 3 *, p<0.05; **, p<0.01.

We independently confirmed the results of RNA-Seq by qPCR. First, we validated the upregulation of eight genes located in the enriched region of chromosome 4, namely *NAF1, RPS3A, ELF2, ABCE1*, *SETD7, NAA15, ZNF827*, and *PPID* (Fig. 4). *NAF1* codes for a box H/ACA RNP biogenesis factor. It is involved in telomere biology and associated with coronary artery disease, pulmonary brosis emphysema, and tumorigenesis^55,56^. *NAF1* was upregulated ~ 2.4-fold in TC4–2A cells. RPS3A codes for a component of the small ribosomal subunit involved in protein synthesis. RPS3A is associated with mild cognitive impairment and Alzheimer’s Disease (AD)[Bibr R57], coronary artery disease[Bibr R58], and hepatocellular carcinoma[Bibr R59]. We confirmed ~ 2-fold increase in RPS3A expression in TC4–2A cells. ELF2 codes for a member of the Ets family of transcription factors and is involved in regulation of B and T cell development, cell cycle progression, angiogenesis and tumorigenesis[Bibr R60]. We observed about ~ 1.7-fold increase in the expression of *ELF2* in TC4–2A cells. *ABCE1* codes for an ATP binding cassette protein that mediates the dissociation of eukaryotic post-termination complexes into free ribosomal subunits. The role of ABCE1 is also indicated in viral infection, tumor proliferation and inhibition of RNase L-mediated apoptosis[Bibr R61]. We captured ~ 1.7-fold increase in the expression of *ABCE1* in TC4–2A cells. *SETD7* encodes an H3K4 histone lysine methyltransferase that can also methylate several other histones and > 30 non-histone proteins[Bibr R62]. Its targets include p53 and several other tumor suppressors and oncogenes, suggesting a role in cancers and development. We confirmed ~ 1.6-fold upregulation of *SETD7* in TC4–2A cells. *NAA15* codes for an auxiliary subunit of the N-terminal acetyltransferase A (NatA) complex. Mutations in *NAA15* are associated with intellectual disability, delayed speech, and autism spectrum disorder[Bibr R63]. We recorded ~ 1.5-fold increase in the expression of *NAA15* in TC4–2A cells. ZNF827 is a zinc finger protein, which is involved in telomere maintenance, homologous recombination[Bibr R64], and modulates the epithelial to mesenchymal transition in breast cancer and cortical development[Bibr R65]. We observed ~ 1.5-fold upregulation of *ZNF827* in TC4–2A cells. PPID belongs to a family of molecular chaperones that regulate protein folding at proline residues, and it is associated with misfolded tau protein highly relevant to neurodegenerative diseases including AD and Parkinson’s Disease (PD)[Bibr R66]. Its expression was found to be increased by ~ 1.4-fold in TC4–2A cells. Interestingly, we noted downregulation of *PPID* in TL4–2A cells, suggesting that C2A-2B-3–4 and L2A-2B-3–4 have opposite effect on expression of *PPID* (Fig. 4).

We validated the C2A-2B-3–4-induced upregulation of six genes located in the right arm of chromosome 7. These were *SSBP1, NOM1, EN2, AGK, REPIN1*, and *NAMPT. SSBP1* encodes a housekeeping protein involved in replication of mitochondrial DNA. Mutations in *SSBP1* are linked to optic atrophy, foveopathy, and Pearson, Kearns-Sayre, and Leigh syndromes^67,68^. Our results showed ~ 1.9-fold increase in expression of SSBP1 in TC4–2A cells (Fig. 4). *NOM1* encodes a nucleolar protein *NOM1* that interacts with eIF4AIII and participates in rRNA biogenesis[Bibr R69]. We observed ~ 1.8-fold upregulation of *NOM1* in TC4–2A cells. *EN2* encodes a homeobox transcription factor. Mutations in EN2 are shown to be associated with autism spectrum disorder[Bibr R70] and overexpression of EN2 is associated with prostate cancer[Bibr R71]. We captured ~ 1.5-fold increase in expression of *EN2* in TC4–2A cells. *AGK* encodes a mitochondrial membrane protein associated with lipid and glycerolipid metabolism. Mutations in *AGK* cause Sefingers syndrome, characterized by congenital cataracts, hypertrophic cardiomyopathy, skeletal myopathy, exercise intolerance, and lactic acidosis[Bibr R72]. *AGK* was found to be upregulated by ~ 1.5-fold in TC4–2A cells. *REPIN1* codes for Replication Inhibitor 1, a zinc finger DNA-binding protein that regulates DNA replication. REPIN1 plays an important role in lipid accumulation in liver and adipose tissue and is a candidate target for treatment of obesity and related metabolic disorders[Bibr R73]. We observed ~ 1.4-fold upregulation of *REPIN1* in TC4–2A cells. *NAMPT* encodes a regulator of intracellular nicotinamide adenine dinucleotide (NAD) metabolism. Ectopic expression of NAMPT is associated with various metabolic disorders and tumorigenesis^74–76^. Expression of NAMPT was upregulated by ~ 1.3-fold in TC4–2A cells, while its expression was reduced to ~ 0.7 times in TL4–2A cells.

We also measured expression of several upregulated genes outside of the enriched genomic regions. *CELSR3* codes for a member of the amingo subfamily, part of the cadherin superfamily. CELSR3, a key component to regulate cell polarity, is required to regulate neuronal axon guidance and wiring in multiple brain regions and neuronal cell types[Bibr R77]. Mutations in *CELSR3* are linked to Tourette’s syndrome[Bibr R78]. We observed ~ 2-fold increase in expression of *CELSR3* TC4–2A cells (Fig. 4). *JADE1* encodes a scaffolding protein that facilitates the interaction of histone acetyltransferase subunit HBO1 with its targets during cell phase transitions[Bibr R79]. We captured ~ 2-fold upregulation of *JADE1* in TC4–2A cells, while its expression was slightly (~ 1.2-fold) increased in TL4–2A cells. *RAC3* is highly expressed in the nervous system and codes for a GTPase that belongs to the RAS superfamily of small GTP binding proteins. Dysregulation of RAC3 has been implicated in intellectual disability and cancer[Bibr R80]. We recorded 2-fold increase in expression of *RAC3* in TC4–2A cells, while its expression was slightly reduced in TL4–2A cells. *CNDP2* codes for a dipeptidase that controls turnover of carnosine and other dipeptides. Dysregulation of *CNDP2* has been indicated in colon cancers, PD, male infertility and obesity^81–84^. Expression of *CNDP2* was increased by ~ 1.5-fold in TC4–2A cells. *IQGAP2* codes for a scaffold protein localized in plasma membrane and involved in cytoskeleton regulation via Rho GTPases. Its involvement in tumorigenesis has been highly investigated[Bibr R85]. We observed ~ 1.25-fold upregulation of *IQGAP2* in TC4–2A cells. *SARS1* encodes a seryl tRNA synthetase. Loss of function of SARS1 is associated with neurodevelopmental delay, deafness, and cardiomyopathy[Bibr R86]. SARS1 was found to be upregulated by ~ 1.25-fold in TC4–2A cells. *ACTN4* codes for alpha-actinin-4, an actin crosslinking protein that is mutated in familial forms of focal segmental glomerulosclerosis, a renal condition characterized by decreased kidney function and scar tissue buildup in the glomeruli[Bibr R87]. *ACTN4* showed a moderate (~ 1.1-fold) upregulation in TC4–2A cells, while it was slightly downregulated in TL4–2A cells. We also measured transcript levels of *SMN1/SMN2*. Interestingly, we captured ~ 1.4-fold increase in expression of *SMN1/SMN2* in TC4–2A cells, while *SMN1/SMN2* expression was reduced by ~ 15% in TL4–2A cells (Fig. 4). Our results suggested that the circular and linear transcripts of *SMN1/SMN2* encompassing exons 2A, 2B, 3 and 4 have positive and negative effects, respectively, on expression of linear transcripts of *SMN1/SMN2*. Of note, qPCR amplification of *SMN1/SMN2* transcripts does not distinguish among different linear isoforms of transcripts generated by *SMN1/SMN2*.

### Validation of a broad spectrum of genes downregulated by overexpression of C2A-2B-3–4

**Figure 5:**
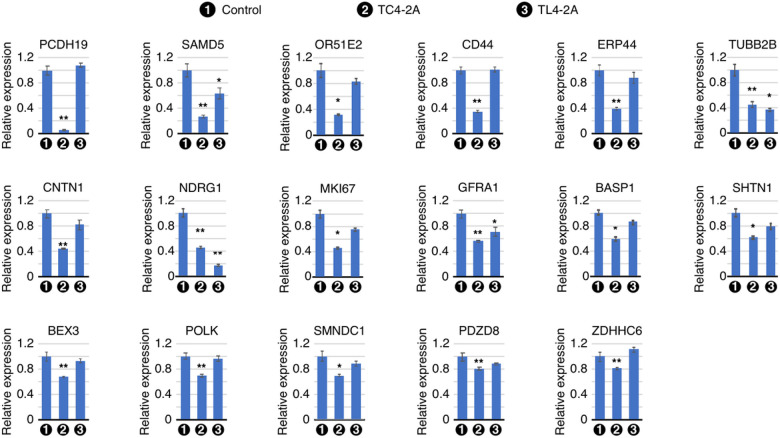
Validation of genes downregulated in RNA-seq. qPCR quantification of mRNA expression levels of significantly downregulated genes upon overexpression of C2A-2B-3–4 is shown in bar graphs. Labeling is the same as in Figure 4.

Employing qPCR, we confirmed the downregulated genes captured by RNA-Seq of transcripts isolated from TC4–2A cells. We began with the validation of six genes located in the enriched region of chromosome 10. These were *MKI67*, *GFRA1*, *SHTN1*, *SMNDC1*, *PDZD8*, and *ZDHHC6* (Fig. 5). *MKI67* codes for a marker of cellular proliferation that is associated with multiple cancers[Bibr R88]. We observed > 55% reduction in expression of *MKI67* in TC4–2A cells (Fig. 5). The expression of *MKI67* appeared to be also reduced in TL4–2A cells, the change was not statistically significant. *GFRA1* codes for a co-receptor for glial cell-line-derived neurotrophic factor (GDNF) and is essential for maintenance of spermatogonial stem cells[Bibr R89]. We captured reduced expression of GFRA1 in both TC4–2A and TL4–2A cells, although the response was slightly strofinger in TC4–2A cells (Fig. 5). SHTN1 codes for SHOOTIN1, an actin-binding protein involved in axon growth[Bibr R90]. We recorded ~ 40% reduction in expression of *SHTN1* in TC4–2A cells. *SMNDC1*, coding for a key protein for spliceosome function, is a *SMN1* paralog. Dysregulation of *SMNDC1* is linked to idiopathic infertility[Bibr R91]. We observed ~ 35% reduction in expression of *SMNDC1* in TC4–2A cells. *PDZD8* codes for an ER protein that tethers mitochondria to the ER in neuronal cells and is essential for endosome recycling^92,93^. ZDHHC6 is a palmitoyltransferase involved in modification of key proteins in ER[Bibr R94]. *PDZD8* and *ZDHHC6* were downregulated by ~ 20% in TC4–2A cells. We validated expression of two X-chromosome linked genes, *PCDH19* and *BEX3*, that were captured by RNA-Seq to be downregulated by C2A-2B-3–4. *PCDH19* codes for a calcium-dependent cadherin with preferential expression in brain. Mutations in PCDH19 are linked to epilepsy, impaired cognitive ability and/or autistic manifestation[Bibr R95]. We confirmed > 16-fold reduction in expression of *PCDH19* in TC4–2A cells, while no effect was observed in TL4–2A cells. BEX3 regulates neuronal survival and differentiation by potentiating transcription of *trkA* gene through the promoter region[Bibr R96]. We observed ~ 35% reduction in expression of *BEX3* in TC4–2A cells.

We validated expression of additional important genes that were captured by RNA-Seq to be downregulated in TC4–2A cells. These were *SAMD5*, *OR51E2*, *CD44*, *ERP44*, *TUBB2B*, *CNTN1*, *NDRG1*, *BASP1*, and *POLK*. *SAMD5* encodes a SAM-domain containing protein of unknown function that has been implicated in multiple cancers^97,98^. We observed ~ 5-fold reduction in expression of SAMD5 in TC4–2A cells, while it was decreased by ~ 40% in TL4–2A cells (Fig. 5). *OR51E2* encodes an olfactory receptor that nonetheless is also expressed in other tissues, particularly in the gut[Bibr R99]. OR51E2 is highly expressed in prostate cancers and regulates their progression[Bibr R100]. We captured ~ 3-fold reduction in expression of *OR51E2* in TC4–2A cells. Protein encoded by *CD44* mediates several functions, including cell-cell interaction, cell adhesion and migration. CD44 acts as a receptor for hyaluronic acid (HA), and serves as a linker between plasma membrane and actin cytoskeleton[Bibr R101]. We recorded ~ 3-fold reduction in expression of *CD44* in TC4–2A cells. *ERP44* codes for the ER protein ERp44, which works as redox sensor and regulates secretion of multiple proteins[Bibr R102]. We observed ~ 40% reduction in expression of *ERP44* in TC4–2A cells. *TUBB2B* encodes a beta tubulin protein that is mutated in polymicrogyria, a cortical development disorder[Bibr R103]. *TUBB2B* was reduced > 2-fold in both TC4–2A and TL4–2A cells (Fig. 5). CNTN1, a neuronal membrane protein, belongs to the immunoglobulin superfamily. Autoantibodies against CNTN1 trigger chronic inflammatory demyelinating polyneuropathy, and CNTN1 is widely considered to be an oncogenic protein in multiple cancers^104,105^. We observed ~ 2-fold reduction of expression of *CNTN1* in TC4–2A cells (Fig. 5). *NDRG1* encodes a universally expressed protein that acts as a tumor suppressor, but it also plays a specific role in Schwann cells and its mutation leads to peripheral neuropathy[Bibr R106]. We observed ~ 2-fold decrease in expression of *NDRG1* in TC4–2A cells. Interestingly, expression of *NDRG1* was reduced by > 5-fold in TL4–2A cells (Fig. 5). BASP1 participates in axonal growth, regeneration, and plasticity. Altered expression of BASP1 has been reported in neurogenerative diseases[Bibr R107]. We captured ~ 40% reduction in expression of *BASP1* is in TC4–2A cells. *POLK* codes for a DNA polymerase catalyzing translesion synthesis in response to DNA lesions. Dysregulation of POLK has been associated with different cancers[Bibr R108]. *POLK* was reduced by > 30% in TC4–2A cells.

### Effect of overexpression of C2A-2B-3–4 on proteome

**Figure 6:**
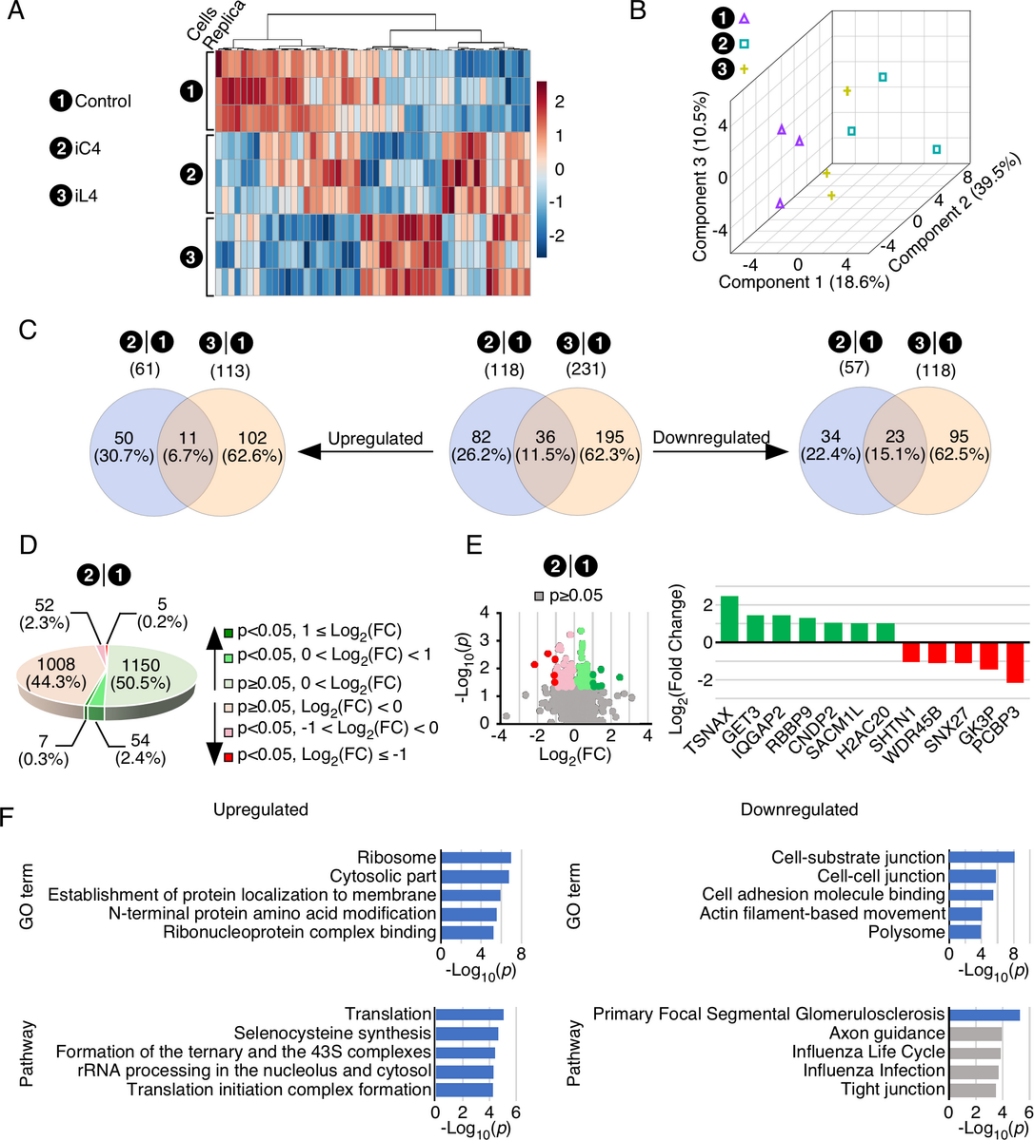
Effect of overexpression of C2A-2B-3–4 and L2A-2B-3–4 on proteome. **(A)** A heatmap of hierarchical cluster analysis of each individual sample of the biological triplicate across three cell lines. The cell lines are indicated on the left side of the heat map. The top 50 proteins are shown. The Euclidean distance was used as similarity measuring parameter, while Ward’s linkage (clustering to minimize the sum of squares of any two clusters) was the clustering algorithm. Each colored square indicates relative abundance of an identified protein. Cell line and replicate was marked on the left of heatmap. On the right side showing a color key (high, red; low, blue) indicates the scale of relative abundance determined by Euclidean distance. **(B)** A three-dimensional (3D) scores plot of Partial Least-Squares Discriminant Analysis (PLSDA) of each individual sample. The top three most discriminant components were selected to plot data in three directions. Each individual replicate is indicated with a colored shape. **(C)** Venn diagrams showing the overlap between genes significantly altered in the TC4–2A and TL4–2A cells compared to control, with overall regulated (center panel), upregulated (left panel) and downregulated (right panel) genes indicated. Two-tailed t test was performed and *p* value <0.05 was considered significant. Cells along with the total number of affected genes are indicated at the top. Of note, there are two proteins (SUB1 (P53999) and YBX3 (P16989)) that were regulated in opposite directions in TC4–2A and TL4–2A, respectively. Hence, the sum of the overlapped subsets of up- and downregulated proteins is 2 less than the one shown in the center panel, while the sum of the non-overlapped subsets is 2 more in both up- and downregulated groups, respectively. **(D)** An overview of proteins identified in TC4–2A (Supplementary Table S4). Proteins are grouped based on regulation direction and statistical significance. Statistical analysis is the same as in (C). The number of proteins identified in each group are indicated, while the percentage of the total identified proteins is shown in parenthesis. Color coding is indicated on the right of pie graph. **(E)** Identification of the most significantly altered proteins in TC4–2A. Left panel: Volcano plot of all identified proteins in TC4–2A. The X axis indicates log2 transformed value of fold change (FC), whereas the Y axis indicates the -log10 transformed p value. Each individual protein was plotted as a colored dot: grey indicates proteins showing p≥0.05, other color-coding is the same as in (D). Right panel: Bar graph including proteins with p<0.05 and log2(FC) ≥1 of expression levels. The protein names are labeled under X axis. Y axis indicates log2 value of fold change (FC). Upregulated and downregulated proteins are shown in green and red, respectively. **(F)** Enrichment analysis (Over-representation analysis, ORA) of significantly upregulated (left panel) and downregulated proteins (right panel) in TC4–2A, respectively. Results of Gene Ontology and Pathway are shown. ER, enrichment ratio.

To profile the changes in the cellular proteome caused by overexpression of C2A-2B-3–4 and L2A-2B-3–4, we performed label-free relative quantification of protein expression. We performed these experiments using exactly the same three groups of cells (control T-REx,

TC4–2A and TL4–2A cells) that were utilized for RNA-Seq. We identified 3818 expressed proteins with an FDR of 0.05. We analyzed the overall composition of the three proteomes using a heatmap of the 50 proteins with the highest divergence between sample groups. As expected, the three groups of cells showed distinctive expression patterns (Fig. 6A). This was further confirmed using principle component analysis (Fig. 6B) The three groups cluster separately, although there were similarities between TC4–2A and TL4–2A cells. After performing data integrity check, filtering, and normalization, we performed differential expression analysis using 2276 and 2279 proteins in TC4–2A and TL4–2A cells by comparing to control T-REx cells, respectively. We identified 118 and 231 significantly altered proteins in TC4–2A and TL4–2A cells, respectively (Fig. 6C–E). Altered proteins were mostly unique to each cell line, however there was significant overlap. Specifically, of 118 proteins that were altered in TC4–2A cells, 82 were unique and 36 were shared with TL4–2A cells, while 1195 proteins were affected in TL4–2A cells alone (Fig. 6C). The proteins upregulated by each group were more distinct, with 50 unique to TC4–2A cells, 11 shared between TC4–2A and TL4–2A cells, and 102 unique to TL4–2A cells. The inverse was true of downregulated proteins with 34 unique to TC4–2A cells, 23 were shared between TC4–2A and TL4–2A cells, and 95 unique to TL4–2A cells.

When we compared TC4–2A cells to control, 61 proteins were significantly upregulated and 57 were significantly downregulated (Figs. 6D,E, Supplementary Table S4). The most significantly upregulated proteins, with > 2-fold change, were TSNAX, GET3, IQGAP2, RBBP9, CNDP2, SACM1L and H2AC20 (Fig. 6E). TSNAX, otherwise known as Trax, functions with partner protein Translin in a complex to activate the RNA-induced silencing complex by cleaving the siRNA passefinger strand[Bibr R109]. TSNAX was the most upregulated protein in TC4–2A cells, increasing by ~ 5.5-fold. GET3 is an ATPase that assists in targeting future tail-anchored membrane proteins to the ER[Bibr R110]. GET3 was the second most upregulated protein with ~ 2.8-fold increase. IQGAP2, a scaffolding protein involved in cytoskeleton regulation, was also captured in our RNA-Seq analysis. IQGAP2 was upregulated by ~ 2.7-fold in TC4–2A cells. RBBP9, a serine hydrolase mainly localized in nucleoplasm, was upregulated by 2.5-fold in TC4–2A cells. CNDP2, a carnosine dipeptidase, was also identified in our RNA-Seq analysis and qPCR validation. The expression of CNDP2 was elevated by ~ 2.1-fold in TC4–2A cells. SACM1L is a phosphatidylinositol phosphatase that plays a role in autophagosome formation[Bibr R111]. SACM1L was upregulated by ~ 2-fold in TC4–2A cells. H2AC20 is a member of the histone H2A family which is one of the core components of nucleosomes. H2AC20 was upregulated by ~ 2-fold in TC4–2A cells.

The five most significantly downregulated proteins in TC4–2A were PCBP3, GK3P, SNX27, WDR45B, and SHTN1 (Fig. 6E). PCBP3 is a KH-domain RNA-binding protein. However, PCBP3 may also bind single-stranded and double-stranded DNA[Bibr R112], and multiple members of the PCBP family including PCBP3 interact with ferritin and iron ions[Bibr R113]. PCBP3 is localized to nucleoplasm, mitochondria, and vesicles, consistent with its multiple roles. PCBP3 was the most downregulated protein, with a 4.5-fold change in TC4–2A cells compared to control. GK3P (also known as GK3) is a glycerol kinase involved in general energy metabolism. GK3P, coded by *GK3*, was downregulated by ~ 2.7-fold in TC4–2A cells. The *GK3* gene is unspliced and located in the intron of another gene, *KLHL2*. Therefore, it may be a pseudogene. Consistently, we did not find many RNA-Seq reads mapping to the *GK3* gene. However, the *GK* gene is predicted to be downregulated ~ 2-fold by our RNA-Seq analysis and qPCR validation (Supplementary Figure S4), while GK3 was upregulated by > 1.7-fold in qPCR validation. It is possible that similarity between peptides produced by GK and GK3P caused the proteomics analysis software to mistakenly assign GK peptides to GK3P. SNX27 is a vesicle sorting protein that mediates recycling of signaling receptors in the brain as well as regulation of glucose and metal ion transporters^114,115^. Low expression of SNX27 is associated with cognitive deficits in Down’s syndrome[Bibr R116]. SNX27 was downregulated by ~ 2.2-fold. WDR45B regulates the maturation of autophagosomes into autolysosomes, and loss of *WDR45B* expression is associated with intellectual disability and other neural phenotypes^117–119^. WDR45B was downregulated by ~ 2.1-fold in IC4. SHTN1 is an actin-binding protein. SHTN1 was downregulated by ~ 2.1-fold in TC4–2A cells. Incidentally, upregulation of *SHTN1* in TC4–2A cells was also captured in RNA-Seq analysis and validated by qPCR.

We analyzed the significantly altered proteins in TC4–2A cells for enrichment of GO terms and KEGG pathways. We focused on top 5 most significant events with FDR < 0.05 (Fig. 6F and Supplementary Figure S1). Consistent with the findings of RNA-Seq, significantly upregulated proteins in TC4–2A cells were associated with translation, ribosome activity, localization of RNA and proteins, and protein modification. Significantly downregulated proteins in TC4–2A cells were mainly related to cytoskeletal activity, cell adhesion, and regulation of extracellular proteins. We also analyzed enrichment of proteins altered in TL4–2A cells compared to control cells and TC4–2A cells compared directly to TL4–2A cells (Supplementary Figures S2, S3). Like TC4–2A cells, TL4–2A cells had upregulation of ribosome components and translation-related proteins (Supplementary Figure S2). However, downregulated proteins were fully distinct.

### Validation of proteins affected by overexpression of C2A-2B-3–4

**Figure 7:**
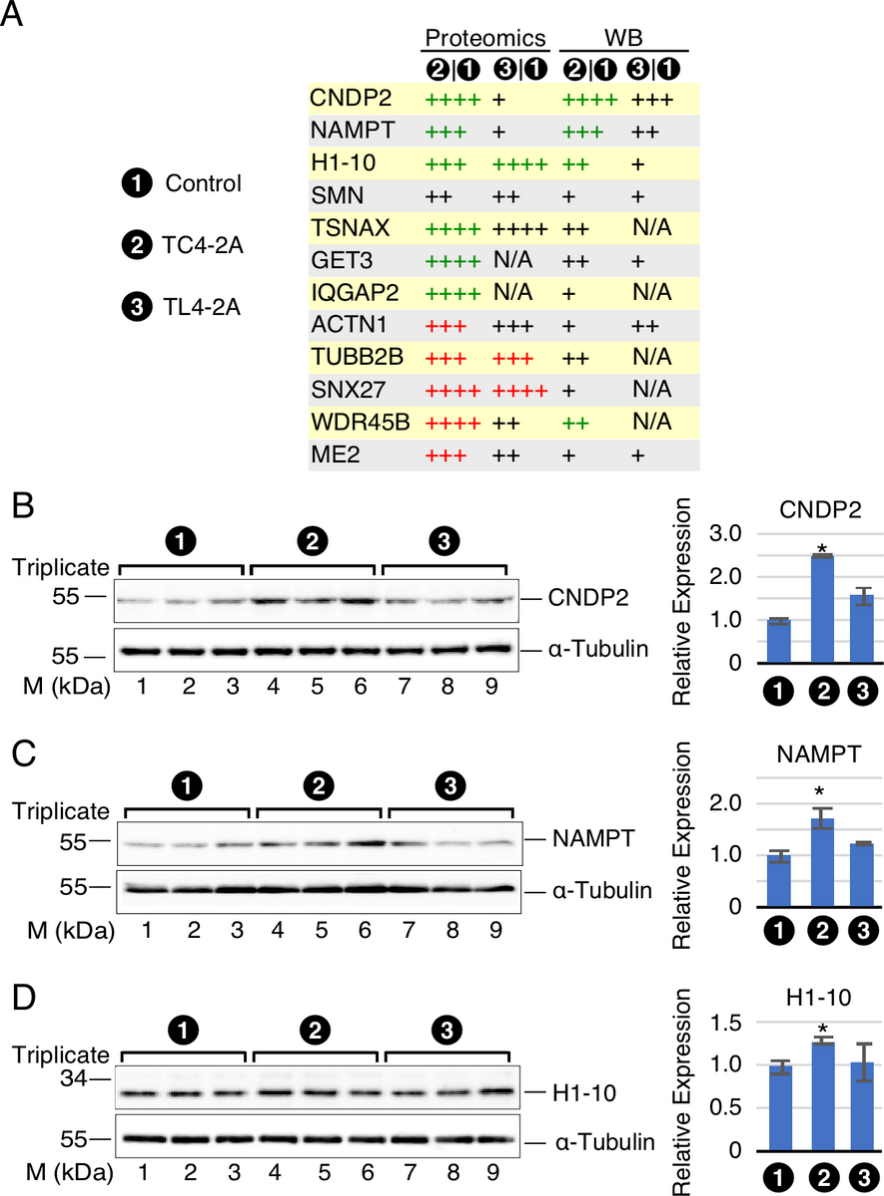
Western blot validation of representative candidates altered significantly upon C2A-2B-3–4 overexpression. **(A)** Expression profiles of top candidates identified in proteomics analysis. Absolute (fold change) <1.2, +; ≥1.2, ++; ≥1.5, +++; ≥ 2, ++++. Green: upregulated, p or adj. p <0.05; red: downregulated, p or adj. p <0.05. **(B-D)** Western blot results of validated candidates. Left panel: Representative blots. α-tubulin is used as loading control. Cell line is indicated at the top of the panel. Antibody used is indicated at the right and nearby molecular weight markers are indicated at the left. Right panel: quantification of western blot results. For each band, background signal was subtracted and then signal was normalized by α -tubulin. Error bars represent standard error of the mean (SEM). n=3, *: p < 0.05compared to T-REx control.

To validate the findings of proteomics, we performed western blot for 14 representative candidates affected in TC4–2A cells (Fig. 7A, Supplementary Figure S4). Our results confirmed the expected upregulation of three proteins, including CNDP2, NAMPT and H1–10 (Supplementary Table S5). For instance, we observed ~ 2.5-fold increase in expression of CNDP2 in TC4–2A cells (Fig. 7B). NAMPT (also known as PBEF) was upregulated by ~ 1.7-increase in TC4 (Fig. 7C). We recorded ~ 1.3-fold increase of H1-10 (also known as H1X) (Fig. 7D). For many proteins, including SMN, we could validate the trend towards upregulation in TC4–2A cells, but the observed changes were not statistically significant (Supplementary Figure S4). This is likely due to the fact that changes captured by proteomics are driven by peptides derived from different protein isoforms not well-recognized by the available antibodies. Also, western blot is not a reliable technique to accurately capture small changes in protein levels. By cross-examining the proteomics and RNA-Seq analysis, multiple candidates, and their coding genes with significantly altered expression were identified in both analyses. These genes included *CNDP2*, *NAMPT*, *IQGAP2* and *TUBB2B* (Supplementary Table S5).

## Discussion

We examined the effect of overexpression of C2A-2B-3–4, a ubiquitously expressed circRNA encompassing exons 2A, 2B, 3 and 4 of *SMN1/SMN2* on the transcriptome and proteome. In parallel, we also examined the effect of overexpression of L2A-2B-3–4, a linear transcript encompassing the entire sequence of C2A-2B-3–4. The objective of our study was to capture the circRNA-specific effects that cannot be exerted by identical sequences in various mRNA isoforms of *SMN1/SMN2*. Overexpression of C2A-2B-3–4 and L2A-2B-3–4 was induced by Dox treatment of HEK293-derived TC4–2A and TL4–2A cells stably expressing C2A-2B-3–4 and L2A-2B-3–4, respectively. We captured a significant impact of C2A-2B-3–4 on transcriptome as expressions of ~ 15% of all detectable genes (4,172 of 26,828 genes) were altered in induced TC4–2A cells. Expressions of 6796 genes were impacted by L2A-2B-3–4. Nearly half of the affected genes by C2A-2B-3–4 were unique as they were not impacted by L2A-2B-3–4. The specific effect of C2A-2B-3–4 could be attributed to multiple factors, including the presence of unique backsplice junction sequence, distinct secondary and tertiary structures[Bibr R120] and m^6^A modifications of circRNAs^121,122^. About 55% of affected genes by C2A-2B-3–4 overlapped with those affected by L2A-2B-3–4. The overlapping effects of C2A-2B-3–4 and L2A-2B-3–4 could be due to common sequence motifs that are not structurally constrained. The specific effects on transcriptome captured with L2A-2B-3–4 but not with C2A-2B-3–4 could be attributed to the structurally unconstrained sequence motifs present exclusively within L2A-2B-3–4.

C2A-2B-3–4 specifically affected large protein-coding genes with higher complexity defined by transcripts with different 5′ untranslated regions (5′UTRs), 3′UTRs and/or alternatively spliced isoforms (Fig. 2). There was a striking enrichment of upregulated genes in the large arms of chromosomes 4 and 7, suggesting that C2A-2B-3–4 promotes formation of extended open chromatin structures in the upregulated regions of chromosomes 4 and 7. Opening of the chromatin structure by C2A-2B-3–4 could be brought about by DNA hypomethylation similarly as reported in case of the exon-containing circRNAs expressed from *FLI1* gene[Bibr R123]. We observed a significant enrichment of downregulated genes in the extended regions of chromosomes 10 and X, suggesting formation of the compact chromatin state in the downregulated regions of these chromosomes. Promotion of repressed state of chromatin may involve factors that favor formation and/or extension of heterochromatin. Expression of genes located on other chromosomes were also impacted by C2A-2B-3–4. Genes that were upregulated by C2A-2B-3–4 were highly enriched for RNA biogenesis and RNA processing factors, especially those associated with the ribosome and spliceosome function (Fig. 3). Downregulated genes were enriched for ER proteins and pathways involved in cell and organ development, morphology, and maintenance of extracellular proteins (Fig. 3). SMN plays an important role in development and maintenance of axonal growth^124–127^. It is possible that the axonal defects observed in SMA could be attributed at least in part to the dysregulation of C2A-2B-3–4.

Among upregulated genes that we validated by qPCR code for important proteins, including H/ACA RNP biogenesis factor (*NAF1*), ribosomal component (*RPS3A*), ribosome associated factors (*ABCE1* and *NOM1*), tRNA synthetase (*SARS1*), transcription factors (*Ets*, *EN2* and *ACTN4*), chromatin modifiers (*SETD7*, *NAA15* and *JADE1*), telomerase associated factor (*ZNF827*), protein folding regulator (*PPID*), mitochondrial factors (*SSBP1* and *AGK*), DNA replication factor (*REPIN1*), NAD metabolism regulator (*NAMPT*), neuronal exon guidance regulator (*CELSR3*), signaling factors (*RAC3*, *CNDP2* and *IQGAP2*). Several alternatively spliced transcripts are generated by *SMN* genes[Bibr R128]. Interesting, *SMN* transcripts were upregulated and downregulated by C2A-2B-3–4 and L2A-2B-3–4, respectively. However, our analysis did not distinguish among different alternatively spliced transcripts of *SMN*. Growing evidence suggest that the expression of genes could be regulated by their own circRNAs through direct recruitment of RNA polymerase (pol II) and/or recruitment of transcription and chromatin remodeling factors as well as by regulation of R-loop formation[Bibr R129]. Such regulation of transcription could have a cascading effect on the nearby genes. Consistently, we captured the effect of C2A-2B-3–4 on transcription of IQGAP2 and POLK situated in the vicinity of *SMN1/SMN2* locus. However, these results should be interpreted with caution as C2A-2B-3–4 is expressed from a different locus in the stable cell line we used. Our finding of upregulation of *SMN1/SMN2* transcripts by C2A-2B-3–4 expressed from a different locus supports a hypothesis that C2A-2B-3–4 serves as a sensor to regulate mRNA levels of *SMN1/SMN2*. Nine of the fteen downregulated genes by C2A-2B-3–4 we validated by qPCR code for factors associated with one or more aspects of neuronal function. These genes were *GFRA1*, *SHTN1*, *PDZD8*, *PCDH19*, *BEX3*, *TUBB2B*, *CNTN1*, *NDRG1* and *BASP1*. Remaining six of the fteen downregulated genes by C2A-2B-3–4 we validated by qPCR code for cancer-associated factors. These genes were *MKI67*, *SAMD5*, *OR51E*, *CD44*, *ERP44* and *POLK*. A recent report showed substantial upregulation *SMN1* transcripts and SMN protein in LSCC[Bibr R34]. It remains to be seen if an increase in *SMN1* transcripts in LSCC is also accompanied with the elevated expression of C2A-2B-3–4.

Various mechanisms may account for the regulation of genes by C2A-2B-3–4. Previous finding that C2A-2B-3–4 is prominently localized in the cytosol may suggest that the effect of C2A-2B-3–4 on transcriptome is exerted through regulation of translation of transcription factors[Bibr R130]. This could be achieved through sponging of specific miRNAs and/or sequestration factors associated with translation. It is also likely that a small fraction of C2A-2B-3–4 remain in the nucleus and modulate transcription of specific genes by interacting directly or indirectly with pol II. SMN is an RNA binding protein with preference for structured RNAs[Bibr R131]. SMN is directly involved in translation of specific mRNAs in neuronal cells[Bibr R132]. SMN also regulates transcription through resolution of R-loops^41,133^. Future studies will reveal if regulation of transcription and translation by SMN is mediated by a C2A-2B-3–4/SMN complex.

We performed label-free proteomics to identify proteins with significantly altered expression in cells overexpressing C2A-2B-3–4 and L2A-2B-3–4. We identified 61 upregulated and 57 downregulated proteins that were affected by C2A-2B-3–4. Majority of the affected proteins were specific to C2A-2B-3–4 (Fig. 6). There was significant overlap in the affected pathways and GO terms between proteome and RNA-Seq analysis. In particular, both data sets suggested an upregulation of ribosomal components and genes/proteins involved in translation, and downregulation of proteins involved in the cytoskeleton and establishment of cell-cell interactions. CNDP2 was the most upregulated protein captured in our validation by western blot. CNDP2 has been implicated in protection of cells against stress-associated conditions[Bibr R134]. With significance to neurodegeneration, an early proteomic study found increased levels of CNDP2 in substantia nigra of PD patients[Bibr R81]. NAMPT was the next most upregulated protein we validated by western blot. Loss of NAMPT has been directly linked to impairment of synaptic vesicles endocytosis/exocytosis at the neuromuscular junction (NMJ) as well as degeneration of hippocampus in mice^135,136^. Considering NMJ formation is impaired in SMA, upregulation of NAMPT by C2A-2B-3–4 will have a direct implication for SMA therapy. H1–10 was the third upregulated protein that we reliably validated by western blot. Given the important role played by H1–10 in chromatin modification[Bibr R137], its upregulation may support a broader role of C2A-2B-3–4 in modulation of gene expression. Although not statistically significant, results of western blot of many proteins, including SMN, supported the trend towards upregulation, consistent with the findings of RNA-Seq and/or proteomic analysis. Further studies will be needed to discern the combined effects of small changes in protein levels due to overexpression of C2A-2B-3–4.

The role of circRNA in gene regulation is an area of growing significance. However, developing efficient tools to uncover functions of circRNAs remain a challenging task. In general, circRNAs and linear transcripts (mRNAs) are produced from the same precursor transcript. Hence, depletion of a circRNA without affecting its linear counterpart remains an arduous endeavor. Production of circRNAs by transient expressions require efficient transfection and does not guarantee sustained expression of circRNAs. In contrast, production of circRNAs using inducible cell lines stably expressing circRNAs provides a better alternative for overexpression studies, although approach is time consuming and limited to specific cell lines. For the obvious advantages, we chose inducible HEK293 cells stably expressing C2A-2B-3–4. The effect of overexpression of C2A-2B-3–4 revealed by RNA-Seq and proteome analysis bring a unique perspective towards our understanding of *SMN1/SMN2* gene function. Many of the functions of C2A-2B-3–4 uncovered here, including transcription, translation and NMJ formation, are dysregulated in SMA. Future studies will reveal if some of functions of C2A-2B-3–4 are shared by other circRNAs such as abundantly expressed C2B-3–4 and as C3–4 generated by *SMN1/SMN2*[Bibr R38]. Carefully designed complementary experiments, including tissue-specific expression and depletion of C2A-2B-3–4 would add to a better understanding of the non-coding functions of *SMN1/SMN2*. While circRNAs of *SMN1/SMN2* were identified ~ 5 years ago, this is the first report on the functions of a *SMN1/SMN2* circRNA. We hope our report serves as a catalyst to invite more investigations into the role of *SMN1/SMN2* circRNAs in processes associated with SMA and other pathological conditions, including cancer and male infertility.
